# Physical Exercise of Primary and Middle School Students From the Perspective of Educational Psychology and Parents’ Entrepreneurship Education

**DOI:** 10.3389/fpsyg.2021.777069

**Published:** 2022-02-10

**Authors:** Chao Song, Sha Ge, Jingjing Xue, Wanxiang Yao

**Affiliations:** ^1^College of Sports Science, Tianjin Normal University, Tianjin, China; ^2^School of Humanities, Beijing Dance Academy, Beijing, China; ^3^Department of Kinesiology, College for Health, Community and Policy, University of Texas at San Antonio, San Antonio, TX, United States

**Keywords:** primary and middle school students, educational psychology, parents’ entrepreneurship education, physical exercise behavior, school education

## Abstract

The study aims to help primary and secondary school students develop a good habit of physical exercise by exploring the current situation of physical exercise of primary and secondary school students and analyzing the factors affecting their physical exercise. Then, intervention strategies are figured out for different groups of students and help them develop a good habit of physical exercise. From the perspective of educational psychology and parents’ entrepreneurship education, the research on the physical exercise of primary and secondary school students is conducted by a questionnaire survey, mathematical statistics and literature review. A total of 280 students from five schools in Tianjin are selected as the research subjects, and the current situation of students’ physical exercise in these five schools is investigated. The results show that 40.5% of boys and 39.4% of girls can take physical exercise more than three times a week; 48.9% of the students do physical exercise for more than 30 mins each time; the students who usually take regular exercise at school account for 82.1%. The physical exercise that students always do is running, badminton and table tennis, which rank the top three among the sports they do. The students usually play basketball, volleyball, and football, and they rarely do the sports like swimming, Wushu, and aerobics. This result is closely related to the characteristics and places of primary and middle school students. The survey shows that the physical exercise awareness of most primary and secondary school students is correct; most students can participate in physical exercise, but few students can do it regularly; parents’ support, parents’ habits of doing exercise, and family’s spending on physical exercise have a significant impact on developing students’ habits of doing physical exercise. Based on the above, it is concluded that the primary and secondary stage is very important for students. Physical education teachers should follow the principle of teaching different students with different methods, enrich teaching materials, and improve teaching quality. The study provides a reference for the reform of PE to guide primary and middle school students to participate in sports activities, improving students’ physical quality.

## Introduction

In recent years, with the continuous reform of school education, society pays more attention to the all-around development of students’ morality, intelligence, and physique. Quality education is becoming the key in school education. In this case, the state issues relevant laws and regulations to comprehensively promote quality education, but their effect is not significant. Although students’ morality and intelligence have improved, their physical quality is declining year by year. It is urgent to enhance students’ physical quality. With the proposal of the “health first” guiding ideology, cultivating students’ physical exercise habits becomes an important task of school physical education. Primary and middle school students are generally in the age of 7–18 years old, and they are in a period of rapid development. Especially with the growth of age, their psychological change is the most complex, the most sensitive, the most volatile, and the school sports, not only for students’ physical quality requirements, but its psychological characteristics are also one of the factors affecting the physical exercise behavior and habits of primary and middle school students. The hobbies of primary and middle school students for sports is gradually shifted from the generalization period to the stable period, and they begin to focus on sports that they are interested in. Using this precious period to guide students’ physical exercise behavior and cultivate their sports habits can achieve twice the result with half the effort.

At present, the research on the physical exercise of primary and middle school students mainly focuses on the influencing factors of students’ physical exercise, like school’s less attention to students’ physical exercise, resulting in the physical education courses being substituted. Based on educational psychology and parents’ entrepreneurship education, a questionnaire survey is conducted and the students from three primary schools and two middle schools in Tianjin are taken as the research subjects. The actual situation of primary and middle school students and their habits of doing physical exercise are discussed. The framework is as follows: the study focuses on the physical exercise awareness, the habits of doing physical exercise, and the influencing factors of primary and middle school students. The questionnaire survey is used to collect data, and the results are analyzed. The innovation is to discuss the physical exercise of primary and secondary school students from educational psychology and parents’ entrepreneurship education. The application of educational psychology to the physical exercise of primary and secondary school students is explored, and the influence of parents’ entrepreneurship education on students’ physical exercise is analyzed.

The study hopes to guide physical education teachers follow the principle of teaching different students with different methods, enriches teaching materials and teaching methods, and improves the teaching quality, improving the physical quality of primary and secondary students. At present, the research on physical exercise of primary and secondary school students shows that the important reason for the decline of physical quality is that they rarely do physical exercise. Therefore, it is necessary to explore a way to strengthen students’ physical exercise and develop their habits of doing physical exercise. The study is helpful to proving the positive effect of parents’ entrepreneurship education, which provides a reference for the development of entrepreneurship education and promotes the development of physical exercise of primary and secondary school students.

## Materials and Methods

### Research on Physical Exercise and Psychology

There are few studies on the relationship between physical exercise and psychological capital in China and foreign countries, and the studies mainly focus on psychological diseases, mental health, happiness, and self-efficacy of college students. Huang and Qiu (2019) studied 428 students’ psychological needs satisfaction, exercise motivation and exercise behavior by using a questionnaire survey, and used three-step mediation regression to analyze the mediating effect of exercise motivation on psychological needs satisfaction and exercise. Ji and Zheng (2021) assessed the relationship between physical exercise and mental health of college students, and found that physical exercise had a positive effect on mental health and social adaptability of college students. In short, the studies on physical exercise and its effect on psychological health is mature. On the basis of previous studies, the investigation is conducted through a questionnaire survey and the characteristics of parents’ entrepreneurship education to help the primary and secondary students develop a good habit of physical exercise.

### Research Samples

According to the content of the study, the stratified sampling and random sampling are combined, and 280 students are randomly selected from five schools in Tianjin as the respondents. There are 105 boys and 175 girls. Among them, 165 are pupils and 115 are middle school students. 300 questionnaires are distributed, and they are all recovered, with a recovery rate of 100%. 280 questionnaires are effective, and the effective recovery rate is 93.33%, which met the research requirements.

### Research Methods

The research samples are investigated and analyzed by a questionnaire survey. The questionnaire is designed by referring to “Taipei high school students’ sports participation questionnaire” compiled by Zeng Ruiyi and “physical exercise questionnaire of adults” by Wuhan Institute of Physical Education. According to the characteristics of primary and secondary school students, the questions in the two questionnaires are re-expressed without changing the original meaning. The samples are selected randomly from five schools in Tianjin and they are asked to complete the questionnaire newly designed. The questions involves their physical states, and the times, intensity, time, place and ways of doing physical exercise. The validity of the questionnaire is tested by experts, and six experts are invited to conduct a five-level comprehensive review and evaluation of the questionnaire. The accuracy of the questionnaire, the integrity, and the rationality of the survey content are tested to ensure that the respondents can fully understand the content of the questionnaire. A total of six validity test questionnaires are distributed, and six are recovered, with a recovery rate of 100%. All six are valid questionnaires, and the effective recovery rate is 100%. Experts’ evaluation of the questionnaire is shown in Tables 1, 2. The tables show that through expert analysis and test, the proportion that the questionnaire meets the survey purpose, tasks, and research needs is more than 80%, which is effective. Combined with the opinions of experts, the questionnaire is modified and supplemented.

**TABLE 1:** Experts’ overall evaluation of the questionnaire content and structure.

	Very reasonable	Relatively reasonable	ordinary	A little reasonable	Not reasonable
Content (%)	29.88	60.00	10.12	0	0
Structure (%)	60.00	29.88	10.12	0	0

*The reliability and validity of the questionnaire are tested. The analysis results show that the Kaiser-Meyer-Olkin (KMO) value of the data in Table 2 is 0.873, and the Bartlett spherical test is P < 0.001, indicating that the reliability and validity of the questionnaire are high and suitable for analysis.*

**TABLE 2:** KMO and the spherical test.

Values		*P*-value
Sampled KMO values	0.873	
Bartlett spherical test		0.000

## Research Results Analysis

### Physical Exercise Behavior of Primary and Middle School Students

In this study, the direct physical exercise behavior of primary and middle school students is mainly reflected in the exercise project, exercise time, frequency, intensity, place, and methods. On the basis of previous studies, this study is conducted from the following aspects.

### Frequency of Physical Exercise

**FIGURE 1:**
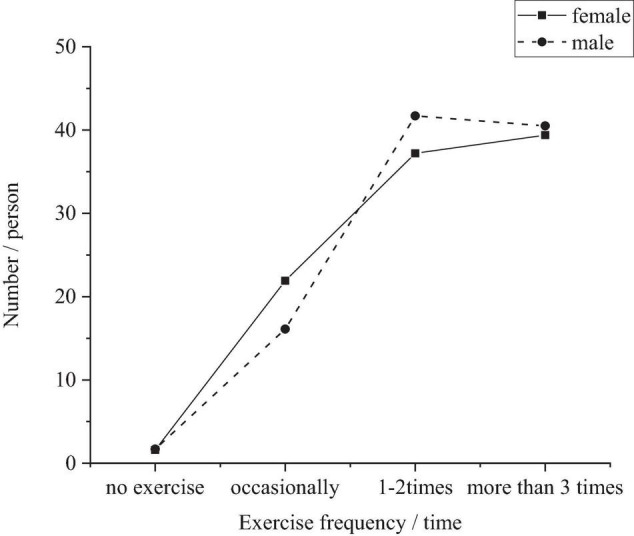
Statistics of weekly physical exercise frequency for boys and girls.

The frequency of physical exercise is the primary indicator to reflect the exercise status, and students are required to have no less than three extracurricular activities per week according to the “Qualified Standards for Physical Exercise of Primary and Middle School Students” (Afolabi et al., 2017). However, Figure 1 shows that only 40.5% of boys and 39.4% of girls take exercise more than three times a week, and the number is less than 1/2 of the total number, and the exercise includes the physical education curriculum arranged by the school. According to the survey, surfing the Internet, watching TV and rest become the main arrangements in their leisure time for primary and middle school students. Nowadays, the disposable time of primary and middle school students is mostly used in entertainment and rest. Especially in today’s rapid development of the Internet, surfing the Internet is the main way of entertainment for primary and middle school students, occupying most of their leisure time. In addition, the high-speed and high-intensity study and life make the students easy to feel tired and unable to engage in physical sports activities (Azzurra et al., 2020).

Therefore, how to carry out relaxation-based sports activities, guide students to participate in, and develop good habits of physical exercise become a focus of research content.

### Physical Exercise Duration

Sufficient physical exercise time is one of the important factors to ensure the effect of physical exercise. The data show that the duration of each exercise should be more than 30 mins to achieve the ideal effect of physical exercise. Table 3 shows that 48.9% of the students can take exercise for more than 30 mins, 27.9% of the students cannot take exercise for 30 mins, and 23.2% of the students do not have a fixed exercise time. That is, a considerable number of the students do not make a reasonable plan according to their physical exercise duration. When the Physical exercise duration is compared between boys and girls, it is found that there is little difference between boys and girls in the arrangement of physical exercise time.

**TABLE 3:** A survey of physical exercise duration of primary and middle school students in Tianjin.

	Not fixed	In half an hour	Half an hour— an hour	1–2 h	Total
Number	65	78	114	23	280
Percentage (%)	23.2	27.9	40.7	8.2	100

### Sports Participating Ways

**FIGURE 2:**
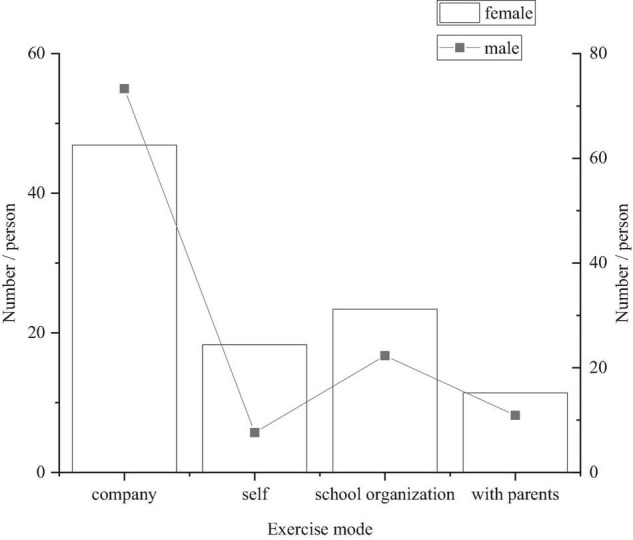
Statistical chart of physical exercise percentage for boys and girls.

Table 4 shows the sports participating ways primary and middle school students with different genders choose. The survey shows that there are certain differences in the choice of sports participating ways between boys and girls in the primary and middle schools. Figure 2 shows that there are a large number of students who exercise spontaneously, reflecting that primary and middle school students have team awareness. However, there are still some students who choose to exercise alone, do exercise organized by schools or with their parents, indicating that the students have a strong dependence on others and should be encouraged to actively participate in physical exercise by schools and families (Allen et al., 2018).

**TABLE 4:** A survey of physical exercises of primary and middle school students in Tianjin.

Sex	Do exercise with others	Do exercise themselves	School-organized exercise	Do exercise with parents	Total
Boys	77	8	12	8	105
Girls	82	32	41	20	175
Total	159	40	53	28	280

### Places of Doing Physical Exercise

**FIGURE 3:**
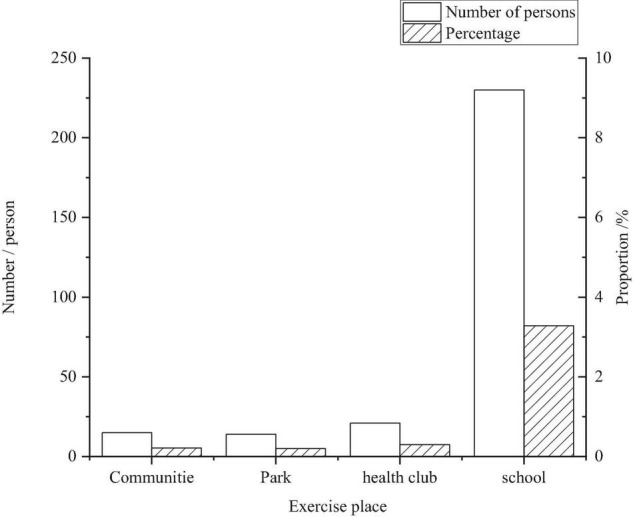
Sites at which primary and middle school students do physical exercise.

The sports site is one of the basic material conditions for physical exercise. The smooth development of many sports projects is closely related to sports sites (Alex et al., 2017). Figure 3 shows that the number of students who take schools as their sites to do physical exercise reaches 82.1%, far more than the community, parks, and fitness clubs. This is in line with the exercise habits of primary and middle school students. The reason is that most of the students at this stage spend their time in schools, and schools become the main places for students to exercise daily naturally (Bendíková and Beáta, 2017). In addition, Table 5 shows that school facilities and exercise equipment are also relatively sufficient and free for students, which is another reason for students to choose schools as the sites for physical exercise.

**TABLE 5:** Primary and middle school students’ satisfaction of the physical exercise site (school).

	No site	Not satisfied	Relatively satisfied	Satisfied	Total
Number	11	58	140	21	230
Percentage (%)	4.8	25.2	60.9	9.1	100

### Physical Exercise Programs

**FIGURE 4:**
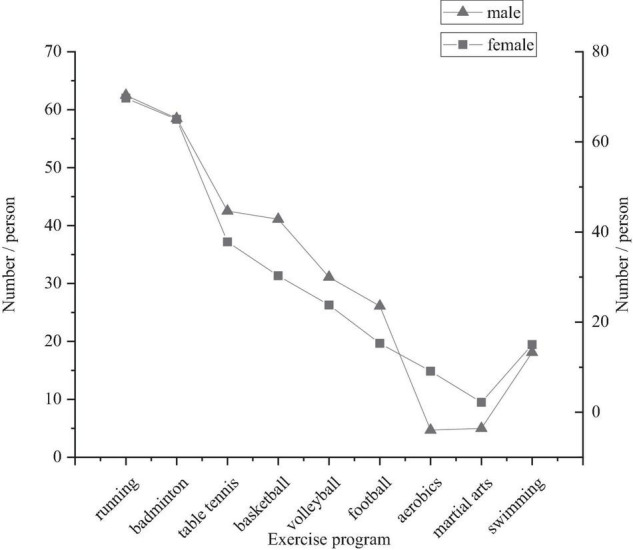
Physical exercise programs of primary and middle school students in Tianjin.

Figure 4 shows that the students in the survey often participate in different physical exercise programs, among which running, badminton, and table tennis account for the highest proportion, followed by basketball, volleyball, and football, and swimming, martial arts, and aerobics takes up the least proportion. This is closely related to the exercise characteristics and exercise sites of primary and middle school students. Figure 3 shows that the main exercise sites of primary and middle school students are schools. In the construction of school sports facilities, the construction of basketball sites and volleyball sites occupies much more land and cost than that of table tennis. In the investigation of the school, it is found that there are more table tennis sites than basketball and volleyball sites. The number of site equipment indirectly affects students’ choice of physical exercise programs (Carina et al., 2018). In the survey of sports programs, primary and middle school students often choose to take the exercise that is easy to carry out, and that there is no special requirement for the site. Many students play badminton in the space of the school and running or fast-walking can be carried out on the path of the campus. Some schools also organize running for the students, which is a simple and effective way to take exercise and is carried out on the campus path (Ennis, 2017).

Figure 4 shows that there are more girls than boys in running, badminton, and aerobics because these programs have less physical contact and weak antagonism. There are more boys than girls in basketball, volleyball, and football because they have high intensity, physical contact, and strong antagonism, which is caused by the physiological differences and personality characteristics between boys and girls.

### Physical Exercise Intensity

The intensity of physical exercise is the load of exercise time, which is often measured by the speed, distance, height, weight, and difficulty of exercise (Boldureanu et al., 2020). The division of exercise intensity in this study is based on the division of physical exercise intensity in the “Adult Physical Activity Behavior Questionnaire” designed by Dr. Duan Yanping and Dr. Brehm of the Institute of Health Science, Wuhan Institute of Physical Education. The types of physical exercise are A: no sweating and no obvious breathing (low intensity) B: slight fever in the body, obvious breathing, and heartbeat (medium intensity) C: a large amount of sweating, shortness of breath, and obvious acceleration of heartbeat (high intensity) (Hornby and Blackwell, 2018).

**FIGURE 5:**
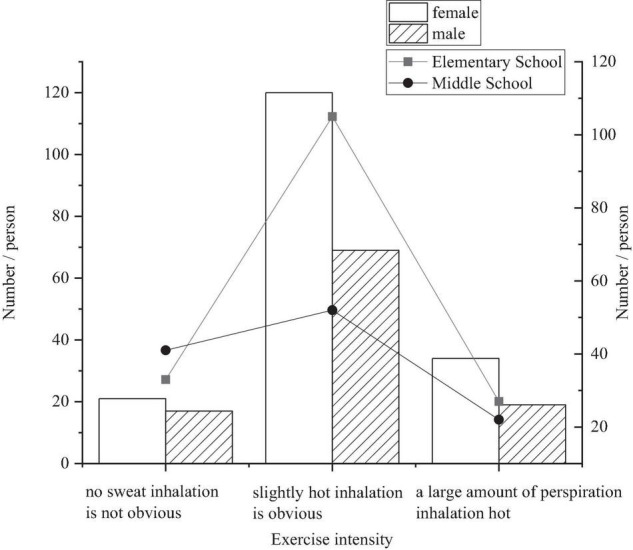
Physical exercise intensity survey of primary and middle school students.

The statistical results in Figure 5 show that according to gender analysis, more students choose an exercise with medium intensity, and boys are more inclined to exercise with high intensity and low intensity. This shows that students’ choice of physical exercise is related to their physical conditions, genders, and personalities.

Middle school students and primary school students preferred moderate-intensity physical exercise, followed by low-intensity exercise, and the number of students choosing high-intensity exercise is less than the other two. The figure shows that the overall intensity of the physical exercise that middle school students choose is significantly higher than that of primary school students.

## Characteristics of Physical Exercise of Primary and Middle School Students

### Psychological Reaction of Primary and Middle School Students in Physical Exercise

**FIGURE 6:**
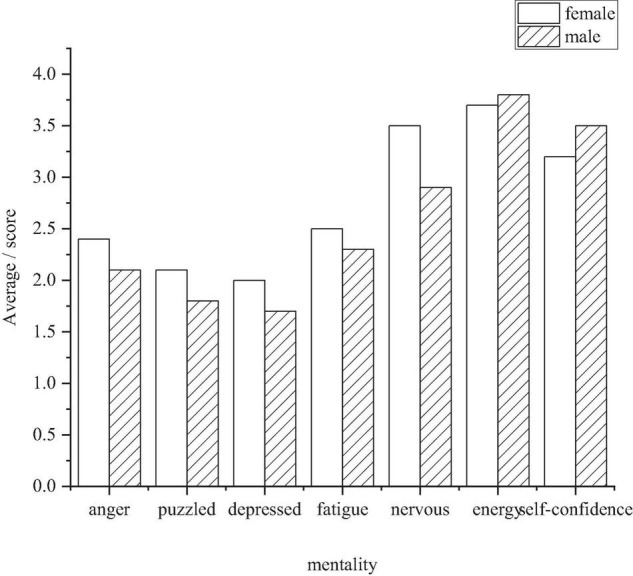
Psychological states of boys and girls participating in physical exercise.

The psychological states of physical exercise of primary and middle school students are studied from the daily exercise activities. The change of psychological states can change with the change of environmental states, or become the transfer of psychological states, that is, the individual in a certain psychological state under the influence of the external environment or under certain conditions into another psychological state (Tilga et al., 2019). Professor Zhu Beili, China, revised the Simplified POMS Scale in 1994, which assessed seven subscales: anger, confusion, frustration, fatigue, tension, energy, and self-confidence. Figure 6 shows the different psychological states of boys and girls (Maynard et al., 2017).

Figure 6 shows that the psychological state of anger, confusion, depression, fatigue, and tension of boys in physical exercise are lower than those of girls, that is, they are stronger than girls in psychological adaptation, and they have more energy and confidence than girls, indicating that boys are more willing to participate in physical exercise.

### Characteristics of Physical Exercise of Primary and Middle School Students

Table 6 shows that the current situation of physical exercise of primary and middle school students in Tianjin presents a trend of small in both ends and large in the middle, and the overall situation of boys is better than that of girls. According to the physical exercise habits, 3.8% of boys and 6.3% of girls participate in physical exercise, and only 22.9% of boys and 9.7% of girls have physical exercise. In the survey, 13.3% of boys and 10.9% of girls are close to the stage of forming physical exercise habits. As long as they insist on doing physical exercise, they are bound to develop a habit of exercise. However, the exercise duration of more than half of the students is not fixed, which is caused by the tense of learning life (Martin and Murtagh, 2017).

**TABLE 6:** Distribution characteristics of physical exercise of primary and middle school students in Tianjin.

Genders		Number	Percentage (%)
Boys	Do not participate in physical exercise, and intend to participate in later	4	3.8
		7	6.7
	There is no fixed exercise time.	56	53.3
	Doing exercise 1–2 times a week	14	13.3
	Doing exercise three times a week, 30 mins each time	24	22.9
		11	6.3
Girls	Do not participate in physical exercise, intend to participate in later	14	8
	There is no fixed exercise time.	114	65.1
	Doing exercise 1–2 times a week	19	10.9
	Doing exercise three times a week, 30 mins each time	17	9.7

### Characteristics of Physical Exercise of the Students of Different Grades

In order to explore the physical exercise of primary and middle school students, the students are divided into three groups according to their participation in physical exercise (Badri and Hachicha, 2019). The first group is the students with physical exercise, namely the students who can carry out regular physical exercise and can ensure the effect of physical exercise. Such students usually do physical exercise more than three times a week for more than 30 mins each time. The second group is the students who can take the initiative to participate in physical exercise, but the effect of physical exercise is not ensured. Such students usually do not spend enough time, have unreasonable exercise intensity, and cannot keep doing physical exercise. The third group is the students who do not participate in physical exercise or who occasionally participate in one or more physical exercises (Sullivan et al., 2017). And a survey is conducted on these three groups of students in different grades, and the detail of the survey is shown in Table 7.

**TABLE 7:** Distribution of physical exercise habits of primary and middle school students in different grades.

	Students with no physical exercise		Students with physical exercise		Students with physical exercise habits		Total	
	Number	Percentage (%)	Number	Percentage (%)	Number	Percentage (%)	Number	Percentage (%)
Students in primary school	35	21.2	78	47.3	52	31.5	165	100
Students in middle school	20	33.3	27	45.0	13	21.7	60	100
students in high school	18	32.7	25	45.5	12	21.8	55	100

Table 7 shows that the number and percentage of the students with physical exercise are the largest in each of the grades. Most of the students can participate in physical exercise, which means that students know about the importance of physical exercise, and they can take action (Scully et al., 2017).

However, the number of students who can form the habit of physical exercise in the survey is very small, accounting for only 31.5, 21.7, and 21.8%, respectively. This phenomenon reflects the problems existing in the process of cognition, action, and habit, namely, the strong sense of cognition and the weak ability to practice. In addition, the tense of learning pressure at this stage prevents the perseverance of physical exercise of students.

### Characteristics of Physical Exercise Behavior Changes of the Students of Different Grades

According to the “stage behavior change model” of Prochaska and Diclemente, Stage 1 is the stage without participating in physical exercise. Stage 2 is the stage in which the students want to participate in physical exercise. Stage 3 is the stage of participating in physical exercise irregularly. Stage 4 is the stage of doing regular physical exercise for less than half a year. Stage 5 is the stage of having an exercise habit.

**FIGURE 7:**
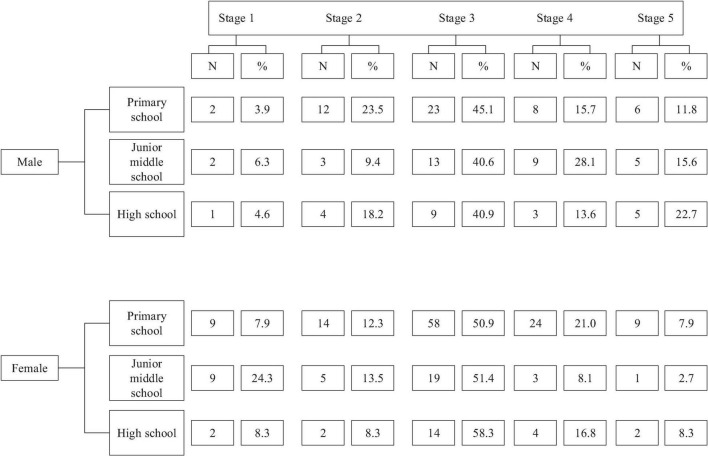
Distribution of the students of different grades at different stages of exercise.

Figure 7 shows that the number of boys in primary schools is 51, that in middle schools is 32, and that in high schools boys is 22. The number of girls in primary schools is 114, 37 in middle schools, and 24 in high school. The number of boys and girls in stage 5 is small, that is, the number of students with physical exercise habits is quite small. In addition, the proportion of boys in stage 5 is higher than that of girls. Combined with the data of stage 5, the reason may be that in the process of education from the lower grade to the higher grade, some students have developed physical exercise habits under the influence of schools, and the number of students in the stage increases. However, with the increase in grades, a large number of students spend a lot of their time on study. And some students who have formed the habit of doing physical exercise daily may have no extra time for physical exercise, and the number of such students fallen back gradually. Stage 5 shows that boys have better physical exercise habits than girls.

Stage 1 tells that the proportion of middle school boys who do not participate in physical exercise is less than that of middle school girls, which may be because girls’ physical fitness is weaker than that of boys, resulting in low enthusiasm for physical exercise.

From stage 1 and stage 2, the number of boys who do not participate in physical exercise in each grade decreases and then increases, which may be due to the low academic pressure of the lower grade and sufficient spare time of the students (Vasconcellos et al., 2020). And physical education curriculum also decreases. In the survey, it is found that in their spare time, sports sites are basically occupied by boys, leaving only track and field runways for girls.

The data shows that the number of students in stage 3 accounts for a large proportion of the five stages. If the spare time is spent in creating opportunities for students to do exercise, more students will take exercise regularly. This plays a positive role in promoting the formation of physical exercise habits of primary and middle school students.

Correct guidance and adequate time are two important factors that can help students form physical exercise habits. The school should increase the control of these two factors, especially enough time. The students in stage 4 do physical exercise, but the duration is short. Schools should give students enough time to actively participate in physical exercise, going forward to stage 5.

## Factors Affecting Physical Exercise of Primary and Middle School Students

### Students’ Factors

Students’ qualities are different, and the effects of the factors affecting physical exercise on them are also different. Many students give up physical exercise because of their laziness and hobbies (Virginia et al., 2017). Interest is a psychological tendency for people to actively know about and explore something or engage in certain activities. For primary and middle school students, their own interest in exercise is an important driving force to support their participation in physical exercise activities (Vodǎ and Florea, 2019). Many students are not interested in participating in physical exercise, but they adhere to the long-term exercise and achieve the effect of physical exercise. The subjects in this study have received formal education and systematic physical education, and they have certain sports knowledge and skills, which is the basis for cultivating their exercise habits. Some students are not willing to personally participate in physical exercise because of their laziness. Also, some students are facing greater learning pressure, they will pay more attention to their study. The neglect of health leads to their failure to put their spirit and mind in participating in physical exercise. The survey results show that the subjective factors are often the main reason for doing exercise regularly (Xiang et al., 2017).

### Family Education Factors

Flobel, a famous German educator, pointed out that parents are the first teachers of children and that families are the first environment for children to grow up in Yilmaz and Soyer (2018). Children are influenced by their parents since there are born. Parents’ ways of speaking and doing things and habits affect those of their children. This study is based on parents’ entrepreneurship education to develop the potential of children.

In the factor education of family education, parents’ physical exercise behavior is often imitated and learned by their children, and greatly affects students’ physical exercise habits. Whether parents can pass on the correct concept of sports and concept to children, and stimulate children’s physical exercise potential will directly affect the students’ physical exercise behavior habits, and is the key to family physical education.

Parents’ entrepreneurship education is also a factor that affects students’ physical exercise. It plays a key role in the development of students’ self-awareness, which is helpful to developing the habit of doing physical exercise. Through entrepreneurship education, students can develop the ability of self-reliance, and cultivate children with the innovative spirit. If they want to achieve their expectations, they need to have a sound multi-quality system. The accumulation of intellectual capital obtained by students in the process of education can help them understand new knowledge. Basic skills in practice can improve students’ ability to identify and create opportunities and do physical exercise regularly, developing their habits of doing physical exercises and improving their physical quality.

## Conclusion

(1) Most of the primary and middle school students’ understanding of physical exercise is correct, and their motivation for physical exercise is physical fitness, stress relief, and interpersonal communication, but a few students think that their doing physical exercise is just for the tests.
(2) Exercises with moderate intensity, such as running, playing badminton, and playing table tennis, and with equal load and weak antagonism and competition are popular among primary and middle school students. Such exercise programs are easy to carry out and can release students’ pressure on study and life. Most students can participate in physical exercise, but the number of students who eventually form physical exercise habits is small.
(3) Parents’ support for physical exercise behavior, parents’ habits of doing exercise, and family’s expenditure for exercise have a significant impact on the development of students’ physical exercise.

Compared with the research results of other scholars, the result of the question “moderate intensity may be more popular with students” is similar to theirs. The reason is that the students in the primary and secondary schools have heavy pressure from study and life, and they don’t have much time to relax themselves. Therefore, many students choose to relieve their pressure through doing the sports with moderate intensity, such as badminton and running.

The current situation of physical exercise of primary and secondary school students in Tianjin is explored, and the main factors influencing their physical exercise are analyzed. Also, students’ interest in doing what kinds of sports is revealed. On this basis, the corresponding intervening strategies are worked out to help them develop a good habit of physical exercise. Doing physical exercise scientifically can improve students’ autonomy and learning effect. It can also enhance students’ physical quality and make them have the awareness of doing exercise lifelong. However, there are some limitations in the study: (1) the questionnaire is subjective, and the students participating in the survey may choose the options that most people will do; (2) the causal relationship and differences between relevant variables cannot be compared in detail, which will be improved in the follow-up research.

## Data Availability Statement

The raw data supporting the conclusions of this article will be made available by the authors, without undue reservation.

## Ethics Statement

The studies involving human participants were reviewed and approved by Tianjin Normal University Ethics Committee. The patients/participants provided their written informed consent to participate in this study. Written informed consent was obtained from the individual(s) for the publication of any potentially identifiable images or data included in this article.

## Author Contributions

All authors listed have made a substantial, direct, and intellectual contribution to the work, and approved it for publication.

## Conflict of Interest

The authors declare that the research was conducted in the absence of any commercial or financial relationships that could be construed as a potential conflict of interest.

## Publisher’s Note

All claims expressed in this article are solely those of the authors and do not necessarily represent those of their affiliated organizations, or those of the publisher, the editors and the reviewers. Any product that may be evaluated in this article, or claim that may be made by its manufacturer, is not guaranteed or endorsed by the publisher.
